# Monitoring the appropriate prescription of low molecular weight heparins and Fondaparinux through administrative data. A retrospective observational study in the Tuscany region

**DOI:** 10.1371/journal.pone.0291628

**Published:** 2023-09-14

**Authors:** Giaele Moretti, Bruna Vinci, Simona Zito, Alessia Caputo, Francesco Attanasio, Milena Vainieri

**Affiliations:** 1 Management and Healthcare Laboratory, Institute of Management, Sant’Anna School of Advanced Studies, Pisa, Italy; 2 School of Specialization in Hospital Pharmacy, Pharmacy Department, University of Pisa, Pisa, Italy; 3 Drugs and Appropriateness Policy Sector, Regional Government, Florence, Italy; University of Turin, ITALY

## Abstract

**Introduction:**

Low Molecular Weight Heparins (LMWHs) and Fondaparinux have been widely used as anticoagulants. Mass prescription may lead to prescriptive inappropriateness, which causes Heparin-induced thrombocytopenia and other side effects.

**Objectives:**

The study investigates the appropriate prescription of LMWHs and Fondaparinux in Tuscany. We aim to validate the crude measure of prescription appropriateness of the Key Performance Indicator (KPI) “Patients treated with LMWHs and Fondaparinux every hundred residents in Tuscany” as a proxy for monitoring prescription appropriateness.

**Methods:**

To compare a crude KPI based only on drug consumption with a refined KPI based on exclusions listed in the clinical guidelines, a retrospective observational cohort study was carried out, using the RECORD guidelines for the year 2019. The refined indicator is computed via record linkage of different datasets regarding (a) pharmaceutical services; (b) hospital discharge records; (c) outpatient services; and (d) birth certificates. We apply exclusion criteria to identify the cohort of patients. Values of the KPI are compared, by ranking, with those obtained from its refined version. A Spearman test was performed to validate the use of the crude KPI as a proxy.

**Results:**

208,717 LMWH and Fondaparinux users are identified, of which 103,299 fall within the study’s inclusion criteria. 16,817 (16%) of LMWHs and Fondaparinux users are classified as high consumption. The refined version of the KPI produces the same ranking results in terms of local health districts (rho = 0.98 p<0.01).

**Conclusions:**

Although the crude KPI is less refined and detailed than the adjusted indicator computed by our study, it has proven capable to provide an accurate snapshot of the use of these drugs across the region. This analysis is useful to enable regional and local managers to run rapid and simple indicators to monitor the appropriateness of LMWHs and Fondaparinux. This analysis should be reviewed periodically to confirm its accuracy.

## Introduction

LMWHs are considered anticoagulants of the first choice, especially compared to standard unfractionated heparin (UFH) [1]. Heparins act by binding antithrombin III, a plasma serine protease inhibitor, thus forming a complex capable of inhibiting several procoagulant serine proteases (factors IIa, IXa, Xa, XIa, and XIIa) [2]. The activity of heparin depends on the length of the polysaccharides which determines the substrate specificity of antithrombin III and on a sequence of pentasaccharides within the molecule. The pentasaccharide sequence alone can determine the inhibition of antithrombin III, specifically on Factor Xa [3, 4]. Heparins show a high affinity for plasma and platelet proteins, endothelial cells, and vascular wall matrix proteins, whose increase in acute phase diseases results in great variability of patients’ response [5, 6] Low Molecular Weight Heparins (LMWHs), which are produced by digestion or depolymerization of Unfractionated Heparin (UFH), have a much lower affinity for this type of protein, show high bioavailability after subcutaneous injection, a predictable and reproducible response [3, 7]. Greater antithrombotic effects, better pharmacokinetics, decreased risk of bleeding, and rapid onset of action are also characteristics attributed to LMWHs [8, 9]. Fondaparinux is a synthetic heparin pentasaccharide that acts directly on Factor Xa and shows no direct activity against thrombin [10]. LMWHs and Fondaparinux are widely prescribed for the prevention and treatment of deep vein thrombosis (DVT), and, in the past few years, for the treatment of patients affected by COVID-19 [11]. LMWHs and Fondaparinux are typically administered in fixed or weight-adjusted doses for therapeutic purposes and thromboprophylaxis. Coagulation monitoring is usually unnecessary, but some authorities suggest it is done in pregnant women, obese patients, and those with renal insufficiency [12, 13]. In recent years, their use has been extended to the prophylaxis of thrombo-embolic events in COVID-19 patients with acute respiratory infections, both bedridden and with reduced mobility worldwide [14]. The rationale for LMWHs use is the need to oppose the clot’s abnormal formation and the platelet hyperactivation determined by the increase in pro-inflammatory cytokines in patients suffering from COVID-19 [15]

In 2021, accordingly, to the National Report on medicines use in Italy (OsMed), LMWHs and Fondaparinux accounted for 31.7% of anticoagulant expenditure, with a noticeable increase compared to the previous year. Therefore, they represent nearly half of the consumption and 27% of the expenditure of drugs used for the treatment of COVID [16]. In Tuscany, they represent 28.7% of total expenditure and about 36.7% of consumption for the anticoagulants class [17] and show a consumption rate 8.6% higher, and an expenditure value 19% lower than the national average.

Considering the impact of this drug class on healthcare costs, policymakers are paying considerable attention to their consumption and expenditure [18]. In addition, there is a need to provide higher-quality service and develop quality measures to assess pharmaceutical care [19]. So, quality measures have been developed to evaluate the services and monitor pharmaceutical expenditure [20].

Hence the importance of developing Key Performance Indicators (KPIs) to understand if economic and health resources are being used correctly. KPIs are important governance tools used to evaluate the efficiency of essential healthcare services and benefits. Each KPI represents a significant tool to support and increase the quality of the decision-making process of regional governments, especially in healthcare where they face multiple barriers and challenges [21]. Indeed, in the last decade, a number of growing studies have been published regarding the use of administrative databases to assess prescription appropriateness [22–24]. The use of administrative healthcare data represented the base for the proposed study. Pharmaceutical care, with the employment of KPIs, can be evaluated on different levels:

- Prescriptive appropriateness, which shows how appropriate a treatment choice is to the patient’s needs and healthcare guidelines. A drug is considered potentially inappropriate when the risk of developing side effects outweighs the benefits, especially if there is clinical evidence supporting an alternative treatment considered to be safer or more effective for that particular clinical condition [25]. Prescriptive inappropriateness may be caused not only by prescription errors (such as prescribing the wrong treatment for a specific pathology) but also by over-prescription. Inadequate knowledge and incomplete information about the patient’s clinical condition and previous treatments can also result in prescribing faults. Prescriptive inappropriateness may increase the risk of developing Adverse Drug Reactions (ADR), which are not only a threat to patient safety but also imply considerable economic costs [26]
- Medication compliance, which refers to the extent of conformity to the recommendations about the dosage, timing, and frequency of medication [27].
- Therapeutic adherence, which is a key factor in treatment success. Adherence to therapies not only affects the patient’s quality of life but also the health care system, as non-adherent patients often need further treatment and hospitalization in worst-case scenarios. The most common consequences include waste of medications, worsening of existing conditions, further need for medications, and increased costs [28].
- Governance of pharmaceutical and medical device expenditures, which indicates an appropriate allocation of resources in the national healthcare system [29].

Since 2005, Tuscany Region has been using a performance evaluation system designed to illustrate and compare, through benchmarking, the different dimensions of the regional healthcare system, including pharmaceutical care [30, 31] Indicators regarding pharmaceutical care are computed using healthcare administrative databases. They are used to compare performance at the national level to understand policy strategies [29]. Additionally, these indicators are used to discern the role that the different levels of health system governance have on the geographic distribution of pharmaceutical prescriptions [32].

Given the recognized importance of KPIs, several institutions and reports propose analyses and risk-adjusted indicators in order to better represent the phenomena mentioned above. This is the case, for example, of Osmed and Aifa, both of which present indicators that can be described as observational studies [16]. In the managerial world, it is not always possible to achieve this level of accuracy, for several reasons: competencies (it is necessary to have staff capable of performing record linkage and analyzing different administrative or clinical data); access to datasets (some levels of government, due to managerial choices, do not have access to all datasets simultaneously); and above all, not all levels of government can perform record linkage. In Italy, the Ministry of Health and Agenas cannot perform record linkages since a decree on the interconnection of administrative streams is still missing. Given the real-world limitation in the managerial practice and the necessity to have “quick and dirty” indicators, this study aims to investigate the appropriate prescription of LMWHs and Fondaparinux through the validation of the crude measure of the KPI regarding the number of patients treated with LMWH and Fondaparinux per hundred residents.

## Materials and methods

To consider the KPI “number of patients treated with LMWH and Fondaparinux per hundred residents” as a good proxy of appropriate prescription of LMWH and Fondaparinux, we compared the results of this indicator in 2019 in Tuscany Region, with the refined indicator calculated through a retrospective observational study on 2019 data in the same Italian region.

In particular, the refined indicator is computed using routinely collected health data, so the REporting of studies Conducted using the Observational Routinely collected Data (RECORD) checklist was applied to improve the quality of reporting [33]. The adoption of the RECORD guidelines was meant at reducing unclear research reports and fostering both transparency and replicability [34]. Information regarding pharmaceutical services provided to non-hospitalized patients can be found in two different datasets: (i) medicines purchased by pharmacies and distributed to patients, (ii) drugs purchased directly by the National Healthcare System and distributed to patients. Information reading the population is provided by the Italian National Institute of Statistics (ISTAT). The population was weighted using Osmed’s Criteria.

The analysis was conducted at the local health district level, i.e. the level of governance that monitors primary care and confronts GPs on the appropriateness of drug prescriptions. Typically, local-health districts have a population of about 100,000 inhabitants. The KPI was computed as the number of patients treated with LMWHs and Fondaparinux over the adult population and includes both appropriate and inappropriate consumption. The refined version of the KPI, instead, is intended to exclude all patients for whom a prolonged use of heparin is appropriate. The exclusion criteria were: patients who had received, up to twelve months before their first prescription, a diagnosis of cancer, venous thromboembolism (VTE), or major orthopedic surgery, such as hip or knee replacement revision, fixing of a vertebral fracture, and hemodialyzed patients. Pregnant women using LMWH or Fondaparinux were also excluded, considering their off-label use to manage high-risk pregnancies.

Data were collected from the regional health information system regarding (a) pharmaceutical services (b) hospital discharge cards; (c) outpatient services; (d) birth assistance certificates. Hospital discharge cards contain data on hospitalizations carried out by public and private hospitals (i.e., patients, dates, and diseases classified with the ICD-9 system). Outpatient services data contain information about services provided in specialist clinics and information about hemodialyzed patients. Birth assistance certificates contain records of all pregnant women giving birth every year in Tuscany. Data were processed using SAS® version 9.4 at the local level, adopting local health districts as units of measurement. Regional administrative data were provided thanks to a collaboration agreement with the Regional Health Service of Tuscany. Administrative data are monitored by the Regional Health Information Office, which anonymizes users by assigning a unique encrypted identifier. The identifier is unique for all administrative data and prevents it from being traced back to patients’ personal data and sensitive information, in compliance with European GDPR 2016/679 and Italian Privacy Law 101/2018. The Italian Data Protection Authority has authorized since 2012, the use of individually collected and anonymized administrative data for research activities without the need to obtain informed consent and approval from the ethics committee [35].

**Fig 1:**
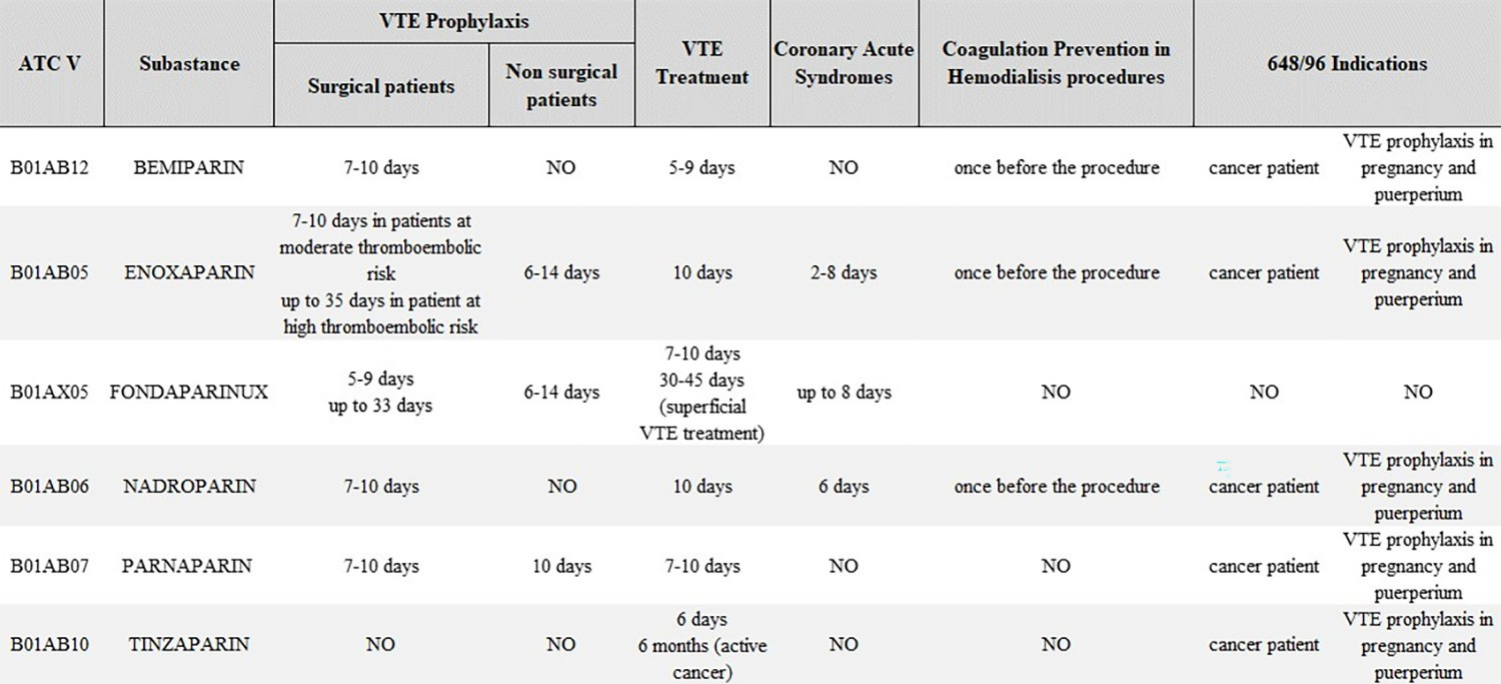
Therapeutical indications per active ingredient.

Medicinal products of the following active ingredient were included in the study: Enoxaparin, Nadroparin, Parnaparin, Bemiparin, and Fondaparinux. Therapeutical indications of each active ingredient were taken into consideration to evaluate prescription appropriateness, as well as the duration of treatment and the number of dosages prescribed to each patient (Fig 1).

Using the patient’s anonymous token as a primary key, data were interlinked at the patient level on the basis of the following inclusion criteria: eighteen or older, resident in Tuscany, and with the first prescription of LMWH or Fondaparinux in 2019.

Prescribing appropriateness can be analyzed through the number of dosage units per patient. Patients who received more than 40 dosage units in the 61 days after the first prescription are considered highly inappropriate. The cut-off of 40 dosage units to consider patients potentially inappropriate was identified by analyzing the Product Information Requirements by Agenzia Italiana Del Farmaco (PIRs) [36–43] for all medicinal products included in the study. As reported in Fig 1, PIRs do not report indications of use longer than 4–5 weeks, except for those already excluded as potentially appropriate. In addition, the cut-off determination was also undertaken by taking into account the different number of posological units contained in each packaging. The refined indicator was computed by taking into account high-consumption users, i.e. those with a consumption of more than 40 dosage units in the 61 days after the first prescription. All users following clinical guidelines and therefore considered appropriate were excluded from the calculation.

Rankings of the two KPIs were compared in order to validate the capacity of the crude KPI to be a good proxy for monitoring inappropriate consumption. We performed a Spearman correlation test between the rankings of both KPIs at the local health district level.

## Results

**Fig 2:**
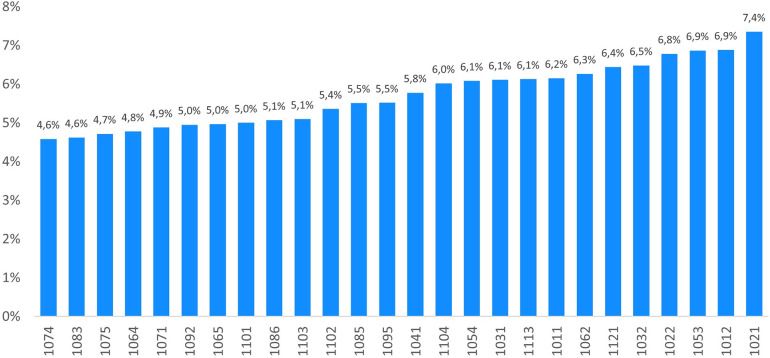
Patients treated with low molecular weight heparins or Fondaparinux per one hundred residents in Tuscany (2019).

The KPI “Patients treated with low molecular weight heparins per one hundred residents in Tuscany” was calculated for the year 2019, at the local health district level. Fig 2 displays the bar charts related to the crude KPI per each local health district. The consumption of LMWH or Fondaparinux ranges from 4.6% to 7.4% among Tuscan users, namely for 208,717 patients in 2019.

**Fig 3:**
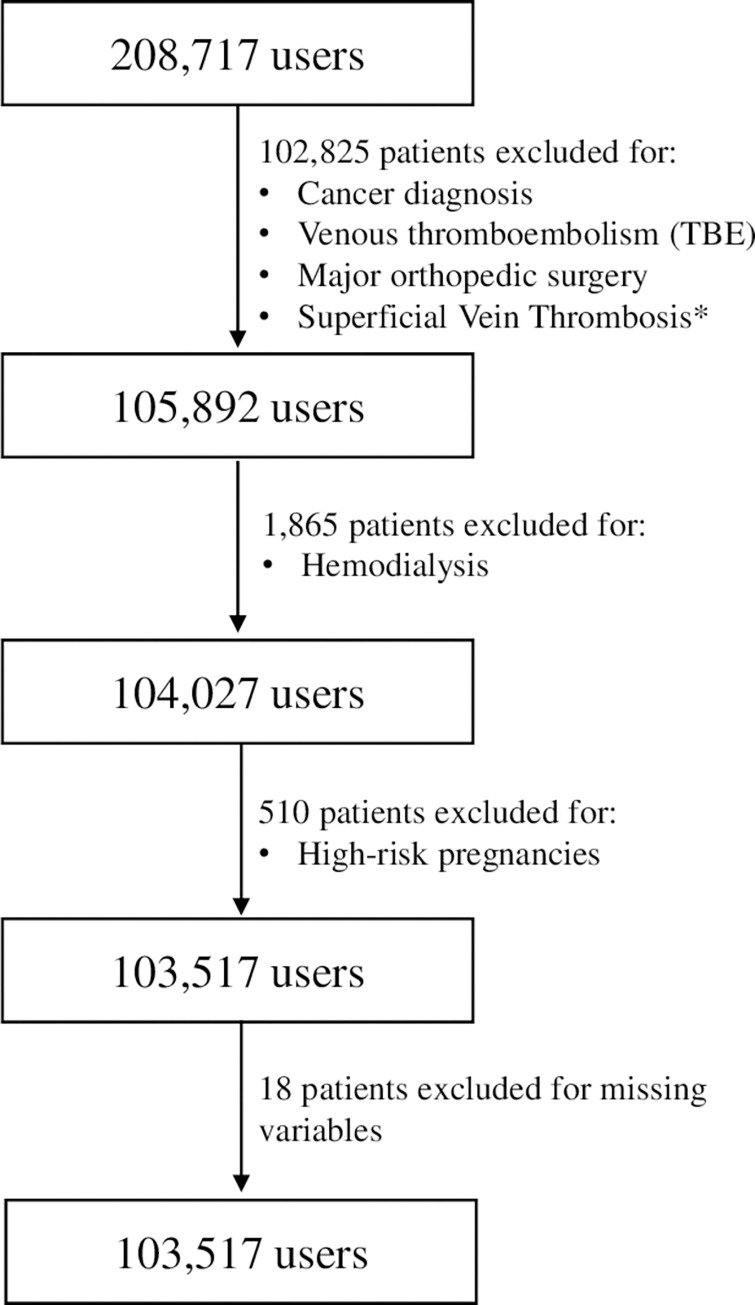
Evolution of the study population in relation to the exclusion criteria.

Fig 3 shows the results obtained from the identification of users from administrative data and the subsequent exclusion procedure. According to the study, 50% of LMWH or Fondaparinux users with a prolonged high consumption (more or equal to 40-unit dosages in 60 days) can be considered appropriate. 102,809 patients received a diagnosis of malignancy, venous thromboembolism, or major orthopedic surgery during the twelve months before the first prescription of LMWH or Fondaparinux. Also, 16 patients with a diagnosis of superficial vein thrombosis and in treatment with Fondaparinux were excluded. 1,865 patients were hemodialyzed, 510 had a potentially high-risk pregnancy and 18 were missing information.

**Fig 4:**
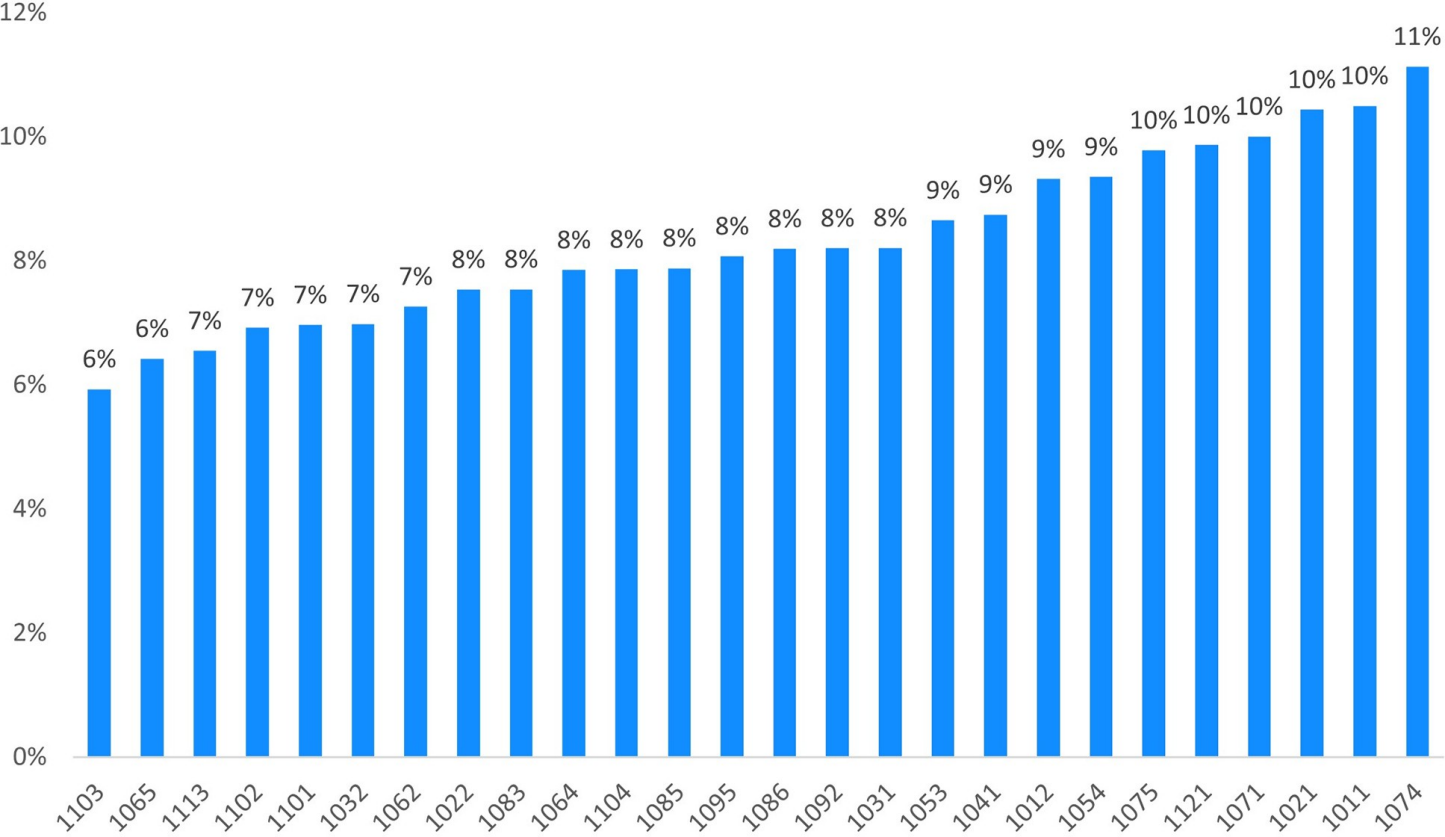
High consumption patients treated with low molecular weight heparins or Fondaparinux on total users.

Of the 103,299 patients meeting the study inclusion criteria, 16,817 were classified as high-consumption users, meaning 16% of the study population. To calculate the refined KPI, only high-consumption users meeting the criteria were considered for the computation. On average, about 8% of the total LMWH or Fondaparinux users in Tuscany are to be considered inappropriate. Fig 4 shows the results of the computation of the refined KPI per local health district.

**Fig 5:**
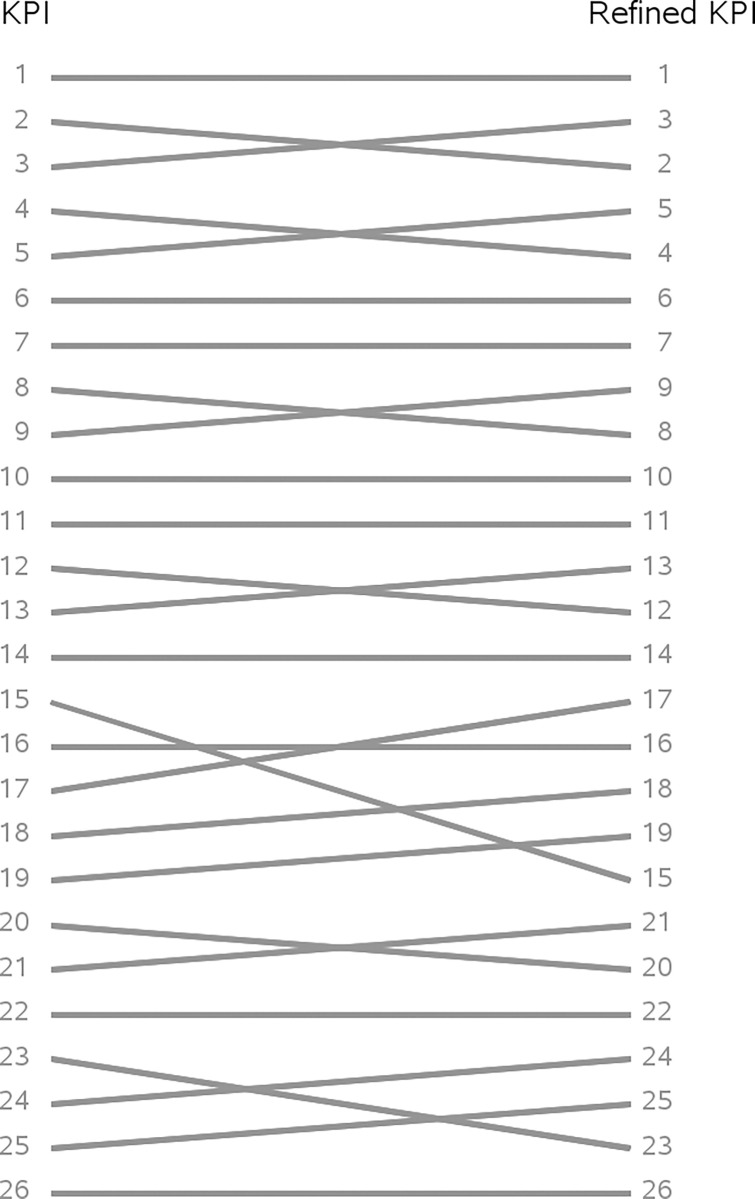
Ranking chart of values from the KPI and its refined version.

Results from the KPI’s refined version were then compared for local health districts, using a ranking chart (Fig 5). The spearman test reported that the ranking of crude KPI is a good proxy of the refined one, rho is 0.98 (p value<0.01).

## Discussion

KPIs are tools used to measure performance trends, to assess how close they are to benchmarks or targets regarding different organizations. This study demonstrated that the crude KPI on LMWH and Fondaparinux consumption, computed by using only the pharmaceutical services dataset, is a good proxy for a refined indicator measuring appropriate prescription. Considering the widespread use of this drug class and its therapeutic importance in the anticoagulant category, the crude KPI is a valuable indicator for monitoring appropriate treatment. In addition, the importance of monitoring these drugs has also increased due to their use in the prophylaxis of thrombo-embolic events in patients with acute respiratory infection caused by Covid-19.

To conclude, we can state that this study validated a crude indicator regarding LMWH and Fondaparinux consumption as a tool to estimate potential prescriptive inappropriateness. The use of this KPI can be easily tracked at both macro/meso (national and regional health system) and micro (local health authority) levels without any specific computational expertise related to record linkage and without the need to build a search protocol.

### Limitations

The calculation of KPI using administrative health data brings some limitations to the study. First of all, pharmaceutical services data only register drug dispensing but cannot provide any information related to drug assumptions by patients. For this reason, the study assumed that prescription could be employed as a proxy for the assumption.

A further limitation is represented by the generalizability of the results. The analyses should be repeated in other contexts to validate the representativeness of the crude KPI against the refined one.

## Conclusions

The results of the study confirmed that inappropriate prescription is more likely to be found in patients who belong to the high consumption users, as there is no indication of prolonged use in patients who do not have one of the conditions represented in the study’s exclusion criteria. Patients for whom prolonged use is recommended represent 50% of the population using LMWHs and Fondaparinux, suggesting a potential inappropriate use in up to 50% of heparin users in Tuscany. The calculation of high-consumption users allowed us to estimate an incidence of possible inappropriate use in 16% of the study population. The new calculation also confirmed the ability of the baseline KPI to provide a good proxy of LMWH and Fondaparinux use in the Tuscany region. The latter, easier to calculate, does not require linkage of multiple administrative databases, and should be considered the reference indicator to monitor the inappropriate consumption of LMWH and Fondaparinux.
