# Correction: Monitoring the appropriate prescription of low molecular weight heparins and Fondaparinux through administrative data. A retrospective observational study in the Tuscany region

**DOI:** 10.1371/journal.pone.0326514

**Published:** 2025-06-13

**Authors:** Giaele Moretti, Bruna Vinci, Simona Zito, Alessia Caputo, Francesco Attanasio, Milena Vainieri

The Funding statement for this article is incorrect. The correct Funding statement is as follows: The Regional Health Authority of Tuscany under a collaboration agreement with Sant’Anna School program funded the research. The views expressed are those of the author(s) and not necessarily reflect those of the Regional Health Authority.
